# Cellulosomics, a Gene-Centric Approach to Investigating the Intraspecific Diversity and Adaptation of *Ruminococcus flavefaciens* within the Rumen

**DOI:** 10.1371/journal.pone.0025329

**Published:** 2011-10-17

**Authors:** Jennifer M. Brulc, Carl J. Yeoman, Melissa K. Wilson, Margret E. Berg Miller, Patricio Jeraldo, Sadanari Jindou, Nigel Goldenfeld, Harry J. Flint, Raphael Lamed, Ilya Borovok, Maša Vodovnik, Karen E. Nelson, Edward A. Bayer, Bryan A. White

**Affiliations:** 1 Department of Animal Sciences, University of Illinois, Urbana, Illinois, United States of America; 2 The Institute for Genomic Biology, University of Illinois, Urbana, Illinois, United States of America; 3 Department of Physics, University of Illinois, Urbana, Illinois, United States of America; 4 Department of Culture Education, Faculty of Science, Meijo University, Nagoya, Aichi, Japan; 5 Microbial Ecology Group, Rowett Institute of Nutrition and Health, University of Aberdeen, Aberdeen, United Kingdom; 6 Department of Molecular Microbiology and Biotechnology, Tel Aviv University, Ramat Aviv, Tel Aviv, Israel; 7 Chair for Microbiology and Microbial Biotechnology, Biotechnical Faculty, University of Ljubljana, Ljubljana, Slovenia; 8 The J. Craig Venter Institute, Rockville, Maryland, United States of America; 9 Department of Biological Chemistry, The Weizmann Institute of Science, Rehovot, Israel; University of Hyderabad, India

## Abstract

**Background:**

The bovine rumen maintains a diverse microbial community that serves to break down indigestible plant substrates. However, those bacteria specifically adapted to degrade cellulose, the major structural component of plant biomass, represent a fraction of the rumen microbiome. Previously, we proposed *scaC* as a candidate for phylotyping *Ruminococcus flavefaciens*, one of three major cellulolytic bacterial species isolated from the rumen. In the present report we examine the dynamics and diversity of *scaC*-types both within and between cattle temporally, following a dietary switch from corn-silage to grass-legume hay. These results were placed in the context of the overall bacterial population dynamics measured using the 16S rRNA.

**Principal Findings:**

As many as 117 *scaC*-types were estimated, although just nineteen were detected in each of three rumens tested, and these collectively accounted for the majority of all types present. Variation in *scaC* populations was observed between cattle, between planktonic and fiber-associated fractions and temporally over the six-week survey, and appeared related to *scaC* phylogeny. However, by the sixth week no significant separation of *scaC* populations was seen between animals, suggesting enrichment of a constrained set of *scaC*-types. Comparing the amino-acid translation of each *scaC*-type revealed sequence variation within part of the predicted dockerin module but strong conservation in the N-terminus, where the cohesin module is located.

**Conclusions:**

The *R. flavefaciens* species comprises a multiplicity of *scaC*-types *in-vivo*. Enrichment of particular *scaC*-types temporally, following a dietary switch, and between fractions along with the phylogenetic congruence suggests that functional differences exist between types. Observed differences in dockerin modules suggest at least part of the functional heterogeneity may be conferred by *scaC*. The polymorphic nature of *scaC* enables the relative distribution of *R. flavefaciens* strains to be examined and represents a gene-centric approach to investigating the intraspecific adaptation of an important specialist population.

## Introduction

All three domains of life inhabit the rumen microbiome, but bacteria numerically comprise the vast majority accounting for up to 10^11^ viable cells per ml [1]. Comparative rRNA sequencing studies have sought to elucidate the phylogenetic landscape and tie specific taxa to the functional potential of the rumen microbiome [2]–[4]. These studies have focused on the entire microbiome, and by design their methods are biased toward the most abundant phylotypes. However, the functional importance of a species to the microbiome does not always correlate with its abundance [5]. In the rumen, specialist cellulolytic bacteria are a clear example. Collectively, major ruminal cellulolytic specialists are found to represent as little as 0.3% of the total bacterial population [6]. Despite their low abundance, deconstruction of cellulose is fundamental to ruminal function. Cellulose accounts for up to 40% of plant biomass and is recalcitrant to depolymerization by most organisms including all mammals. In addition, the intimate associations cellulose forms with other structural polymers, such as hemicellulose, pectin and lignin make its deconstruction critical to the effective use of these materials [7].

*Ruminococcus flavefaciens* is a specialist cellulolytic bacterial species characterized from the rumen, other herbivorous animals and humans [1], [8], [9]. Currently *R. flavefaciens* is the only rumen bacterium known to produce a defined cellulosome [10]–[12]. The synergism imparted by the concerted action of fibrolytic enzymes that assemble as cellulosomes is usually associated with improved cellulolytic efficiency [13]. The scaffoldin of the *R. flavefaciens* cellulosome is assembled from four protein components (ScaA, ScaB ScaC and ScaE) that are encoded from a single gene cluster (the *sca* cluster). ScaA and ScaB are considered the primary scaffoldin proteins that incorporate the various enzymes into the complex, whereas ScaC is thought to act as an adaptor enabling variable configurations of the cellulosome. ScaE appears to have a role in attaching the cellulosome to the bacterial cell surface [14].

Isolation and *in vitro* characterization studies of different *R. flavefaciens* strains have revealed remarkable sequence and functional diversity among the strains, including their ability to break down cellulose [10], [11], [15]–[17]; yet it is not clear if this diversity manifests within an ecological context. Although the gene organization of the *sca* cluster appears to be identical among strains (*scaC-scaA-scaB-cttA-scaE*), the DNA sequences and resulting structural arrangement of the modular components are quite divergent, suggesting a further level in intraspecific diversity of cellulosome organization over the adaptable configurations available [10].

Previously we have shown the scaffoldin gene, *scaC*, to be a powerful tool in discriminating strains of *R. flavefaciens* [11]. ScaC is a small dockerin- and cohesin-containing scaffoldin that binds both to ScaA via its dockerin and a range of yet-to-be-identified polypeptides via its cohesin [18]. To date the gene encoding ScaC has only been found in strains of *R. flavefaciens*.

In the present work, we employed terminal restriction fragment length polymorphism (T-RFLP) to gauge community structure and dynamics in three rumen microbiomes using both the 16S rRNA and *scaC* genes over a period of six weeks following a dietary switch from corn-silage to grass-legume hay. We separated samples into liquid and solid fractions to evaluate differences between planktonic and fiber-associated communities and sampled both one and nine hours post-feeding to ascertain any effect of the availability of fresh substrate. These analyses were supplemented with the generation and examination of an ∼1100 *scaC* clone library.

## Results

### T-RFLP Analysis of Rumen Bacterial Community Composition and Dynamics

**Figure 1:**
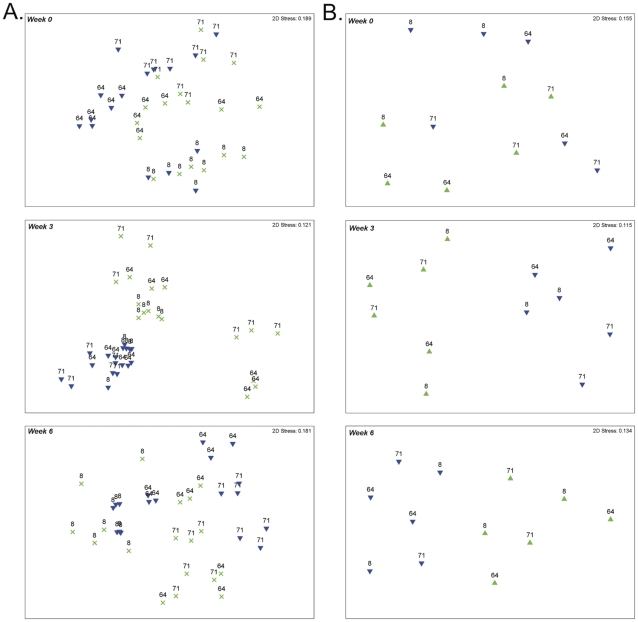
NMDS plots of 16S rDNA and *scaC* T-RFLP analyses. Plots are generated from the restriction enzymes with the most consistently significant p-values (*Msp*I for 16S (A) and *RsaI* for ScaC (B)). Weeks are indicated at top left of each panel. Numerical representations indicate bovine number (8, 64, or 71) and symbols indicate either fiber-adherent fraction (green **×**) or liquid fraction (blue ▾).

The rumens of three cattle (8, 64, and 71) were sampled both one and nine hours post-feeding at 2, 5 and 8 weeks (sampling weeks 0, 3 and 6) following a dietary switch from a corn-silage to a medium-quality grass-legume hay diet. Samples were separated to give fraction-specific information for the fiber-adherent and planktonic microbes. To evaluate the plasticity of the rumen microbiome between individual cows, between fractions and temporally between time post-feeding and weeks post-dietary switch, we employed T-RFLP of the 16S rRNA gene using three different restriction enzymes (REs; *AluI*, *HhaI*, and *MspI*). We constructed Bray-Curtis similarity matrices of square root transformations for all samples. Each variable was evaluated for its potential contribution to the observed dissimilarity among samples. At week 2, the majority of the dissimilarity observed was attributable to differences between the individual cows sampled regardless of RE tested (p<0.01; Figs. 1a, S1 and Table 1). As the study progressed however, a greater proportion of the dissimilarity was explainable by temporal differences and differences between fractions (pertaining to planktonic or fiber-adherent communities). Differences between planktonic and fiber-adherent fractions were the most discriminating factor at week 5 (p<0.01). Of all metrics tested, time post-feeding described the least amount of variation between samples. While, aside from week 5, variation between individual cows was the largest discriminating feature (p<0.01; Table 1).

**Table 1:** 16S rRNA ANOSIM results.

		*Alu*I		*Hha*I		*Msp*I	
Week	Factors	R-value	p-value	R-value	p-value	R-value	p-value
2	Individual	0.497	**<0.01**	0.247	**<0.01**	0.746	**<0.01**
	*Pairwise (8, 64)*	0.412	**<0.01**	0.179	**<0.01**	0.778	**<0.01**
	*Pairwise (8, 71)*	0.67	**<0.01**	0.368	**<0.01**	0.855	**<0.01**
	*Pairwise (64, 71)*	0.372	**<0.01**	0.234	**<0.01**	0.616	**<0.01**
2	Hour Sampled	0.002	0.41	0.024	0.18	0.074	0.06
2	Fraction	0.109	**<0.01**	0.187	**<0.01**	0.185	**<0.01**
5	Individual	0.273	**<0.01**	0.223	**<0.01**	0.216	**<0.01**
	*Pairwise (8, 64)*	0.307	**<0.01**	0.213	**<0.01**	0.291	**<0.01**
	*Pairwise (8, 71)*	0.318	**<0.01**	0.329	**<0.01**	0.315	**<0.01**
	*Pairwise (64, 71)*	0.197	**<0.01**	0.144	**<0.01**	0.083	**<0.01**
5	Hour Sampled	0.084	**0.04**	0.040	0.12	0.077	**0.04**
5	Fraction	0.507	**<0.01**	0.043	**<0.01**	0.542	**<0.01**
8	Individual	0.333	**<0.01**	0.391	**<0.01**	0.602	**<0.01**
	*Pairwise (8, 64)*	0.347	**<0.01**	0.380	**<0.01**	0.622	**<0.01**
	*Pairwise (8, 71)*	0.532	**<0.01**	0.603	**<0.01**	0.836	**<0.01**
	*Pairwise (64, 71)*	0.139	**<0.01**	0.167	**<0.01**	0.299	**<0.01**
8	Hour Sampled	0.133	**<0.01**	0.107	**0.01**	0.130	**0.01**
8	Fraction	0.236	**<0.01**	0.213	**<0.01**	0.183	**<0.01**

R-coefficients and p-values were calculated for factors (individual, sampling hour, or fraction) across all weeks sampled and compared across T-RFLP restriction enzymes at 10,000 permutations An R-coefficient of 0 indicates complete similarity while 1 indicates complete dissimilarity. A p-value≤0.01 or ≤0.05 is significant (as indicated in bold font).

To gain a broad insight into the taxonomic structure of the microbiomes, we compared the T-RFLP patterns to *in silico* RE digests of full-length rumen 16S rRNA libraries [3] using the web-based phylogenetic assignment tool (PAT; Table S1; [19]). Between 81% and 89% of all T-RFLP fragments matched to *in silico* fragments present in the 16S rRNA fragment library [3]. The fragments were largely assigned to the phylum *Firmicutes* (26%–45%), with smaller contributions from the *Bacteroidetes* (1%–4%), *Proteobacteria* (<1%), and TM7 (<1%). These observations were consistent with our previous gene-centric metagenomic analysis of the week 8 samples [3]. The remaining 54%–74% of the fragments were unable to be classified.

Although we were unable to elucidate the taxonomic break-down beyond the phylum level, our previously reported metagenomic-based analysis of the week 8 samples found members of the *Ruminococcus* genera to be rare in both planktonic and fiber-adherent fractions from each animal [3].

### T-RFLP Analysis of Rumen ScaC Community Dynamics

To determine if the temporal dynamics of *R. flavefaciens scaC*-types followed that observed for the overall bacterial community, we examined community *scaC* profiles using T-RFLP. The *scaC* gene was amplified from the same rumen metagenomic samples as used for analysis of the 16S rRNA gene, for *scaC* amplicons digestions were performed using the REs *AluI*, *HaeIII*, *MspI*, and *RsaI*. Bray-Curtis similarity coefficients and Non-metric Multidimensional scaling (NMDS) plots were again generated for all samples.

Breaking down the relative contributions of each variable to the patterns observed through analysis of similarities (ANOSIM) revealed a similar dynamic of the *scaC* profiles to that observed for the community 16S rRNA over the first two sampling weeks (Table 2). Initially, the R-statistic suggested differences between individual cows accounted for the most dissimilarity observed between samples although p-values (p = 0.06–0.51) indicated little significance. As the study progressed R-values for differences between fractions increased, analogous to that observed for the 16S profile. Similarly, at week 5 differences between planktonic and fiber-adherent fractions appeared the most significant factor and here the R-statistic was supported with p-values as low as p = 0.002. However, unlike the dynamic observed for the overall bacterial community, differences between fractions remained the most significant factor at week 8, again being supported by strong p-values (as low as p = 0.002). Unlike the 16S profile, at week 8 no significant difference was seen in *scaC*-types among animals (Figs. 1 and S2; Table 2). ANOSIM analysis of the temporal variable ‘hours post sampling’ almost always (10 of 12 analyses) resulted in a negative R-statistic indicating greater variation within the sample group than between sampling times.

**Table 2:** *scaC* ANOSIM results.

		*Alu*I		*Hae*III		*Msp*I		*Rsa*I	
Week	Factors	R-value	p-value	R-value	p-value	R-value	p-value	R-value	p-value
2	Individual	−0.014	0.51	0.167	0.17	0.257	0.06	0.273	0.06
2	Hour Sampled	−0.093	0.82	−0.083	0.72	0.109	0.21	0.143	0.15
2	Fraction	0.08	0.21	0.104	0.24	0.017	0.40	−0.011	0.50
5	Individual	0.204	0.06	0.167	0.09	0.169	0.08	0.009	0.39
5	Hour Sampled	−0.035	0.60	−0.12	0.89	−0.089	0.81	−0.063	0.65
5	Fraction	0.226	**0.04**	0.111	0.16	0.267	**0.03**	0.859	**0.002**
8	Individual	0.079	0.25	−0.213	0.93	0.076	0.27	−0.137	0.83
8	Hour Sampled	−0.128	0.90	−0.027	0.54	−0.096	0.81	−0.137	0.86
8	Fraction	0.041	0.34	0.364	**0.02**	0.239	**0.04**	0.743	**0.002**

R-coefficients and p-values were calculated for factors (individual, sampling hour, or fraction) across all weeks sampled and compared across T-RFLP restriction enzymes at 999 permutations.

Similarity percentage (SIMPER) calculations comparing planktonic and fiber-adherent fractions by sample week revealed two to three *scaC* fragments (depending on RE used) that were consistently within the ten most discriminating bands throughout the sampling period (Table S2). This indicates that the *scaC*-types with which these fragments were associated were significantly and consistently enriched in one or other of the sample fractions. Similarly, SIMPER analysis of the 16S rDNAs between planktonic and fiber-adherent fractions identified three to four fragments that were consistently within the ten most discriminating bands (Table S3).

### Evaluating the Extent of ScaC Diversity

We next sought to determine the extent of *R. flavefaciens* strain diversity inherent within a rumen microbial ecosystem. To achieve this, we cloned and sequenced 1106 *scaC* genes obtained through PCR amplification of our rumen microbiome samples. Clones were evenly sampled from liquid- (n = 558) and fiber-extracts (n = 548), from each of the three rumens (n = 401, 322 and 383 for rumens 8, 64 and 71, respectively) and at both one (n = 551) and nine (n = 555) hours following feeding.

Using FastGroupII [20], we binned the *scaC* nucleotide sequences at varying levels of sequence similarity. The number of bins (corresponding to *scaC*-types) decreased from 281 to 51 as we reduced the clustering threshold from 99% to 40% identity (Table 3). Plotting the clustering threshold against the resulting number of groups produced a sigmoidal curve that plateaued between the clustering thresholds 70% and 50%. Therefore to gain information on the abundances of *scaC*-types both within and between individual cows, we selected a 70% clustering threshold as it represented the most discriminating point of the plateau. To ensure the adequacy of this clustering threshold, we aligned the protein translation of all sequences and examined their conservation within and between clustered *scaC*-types (Fig. S3). Even at the protein level each *scaC*-type was clearly distinct from all others. Further, each *scaC*-type displayed strongly conserved regions that were unique among the collection (Fig. S3).

**Table 3:** Clustering statistics for *scaC* at various sequence identity thresholds.

	Clustering threshold						
	99%	90%	80%	70%	60%	50%	40%
Number of valid sequences	1106	1106	1106	1106	1106	1106	1106
Number of groups observed	281	141	95	75	70	67	51
Chao1 total diversity estimate	1285.9	301	199.7	117.7	106.5	104.6	67.0
Shannon-Wiener index	4.186	3.5675	3.2294	3.1149	3.0984	3.0672	2.5746

**Figure 2:**
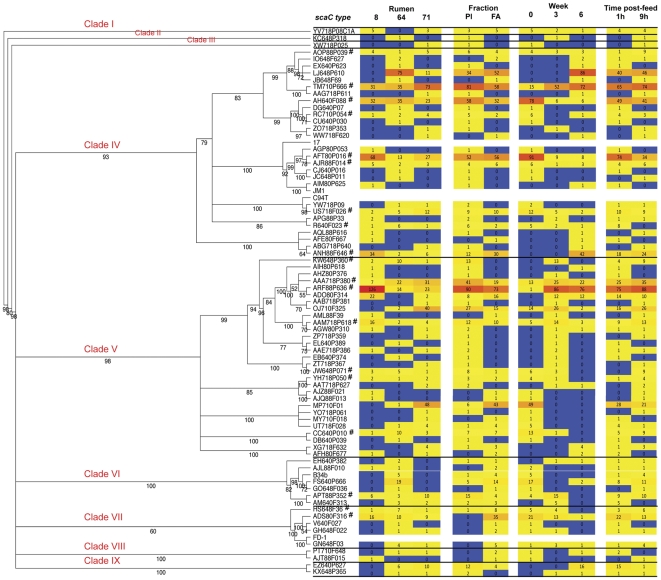
Maximum likelihood tree showing the phylogentic relationship of the *scaC*-types alongside a heatmap of their relative distribution among sample sets. Bootstrap values and clade distinctions are shown on the tree branches. The heatmap is aligned to the tree and shows the actual number of observations of each *scaC*-type and is colored in shades from blue (n = 0) to yellow (50 percentile) to red (maximum). The *scaC*-types are indicated and branch tips and universal types are denoted with ‘#’. The positions of reference strain *scaC*'s are also shown.

Using the 70% clustering threshold, we identified 75 distinct *scaC*-types from all clones sequenced (Tables 3 and S4). Maximum likelihood analysis of the 75 *scaC*-types, along with those from reference strains using RaxML [21] revealed a broad phylogeny with nine deep-rooted clades (Fig. 2). Non-parametric Chao1 estimators using DOTUR [22] predicted that at this clustering threshold as many as 117 *scaC*-types may have been present among the rumens sampled (Table 3). For individual rumens Chao1 predictions ranged from 68 (rumen 64) to 107 (rumen 71; rumen 8 was predicted to have 76) *scaC*-types.

*R. flavefaciens* strain B34b was the only one of the several reference strains whose *scaC* we had previously sequenced [11] that clustered with any of the metagenomic *scaC* sequences. The *scaC* of strain B34b clustered with five other sequences. Lower clustering thresholds did not improve this finding dramatically, although strain C94T binned with 8 other sequences from a clustering threshold of 60%.

**Figure 3:**
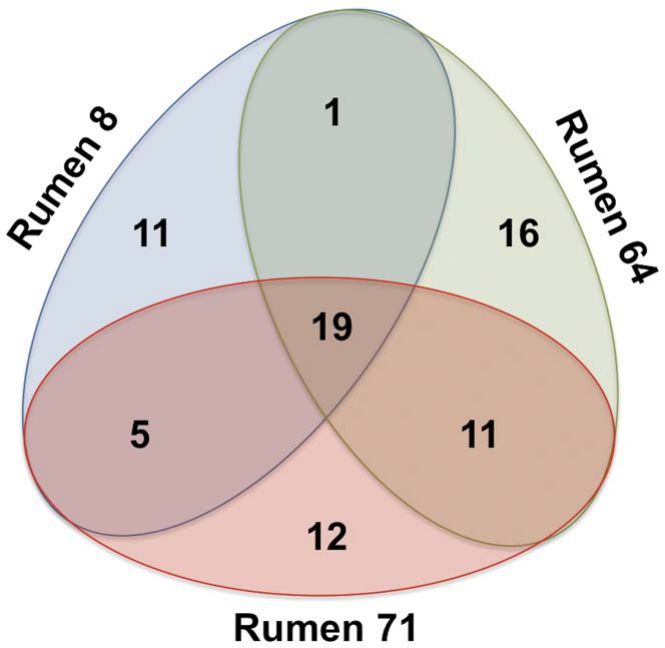
Venn diagram showing the relative occurrence of *scaC*-types in the three rumen samples.

We observed variation between individual animals with just 19 of the *scaC*-types being detected in each of the three rumens sampled (Figs. 2 and 3). The B34b *scaC*-type was not observed in rumens 8 or 71 (Fig. 2 and Table S4). There was also variation in the relative abundances of the various *scaC*-types. For instance, the most predominant group (ARF88P636) accounted for 31% of all *scaC*-types from rumen 8, but only 4–6% of the *scaC*-types from rumens 64 and 71. The 19 *scaC*-types found in all three rumens (henceforth referred to as the universal *scaC*-types) collectively accounted for the majority of sequences obtained from each rumen (89%, 58%, and 62% for rumens 8, 64, and 71, respectively; Table S5).

Certain universal groups were enriched in one or other of the sample fractions (planktonic or fiber-adherent). Group ADS80F316 (n = 35) was found exclusively in fiber-adherent samples, while group KW648P360 (n = 13) was found exclusively in the planktonic samples (Fig. 2 and Table S6). There appeared to be some trend associating *scaC*-types being enriched in planktonic or fiber-associated fractions and their phylogeny. For instance, *scaC*-types from clade VII, which included the universal types HS648F36 and ADS80F316 and included the type strain FD-1, and clade VIII were only detected once in the planktonic phase but 54 times in the fiber-associated fraction. Certain regions of the larger clades (clades IV and V) were more frequently found in the planktonic phase (Fig. 2).

A clear temporal pattern was observed among *scaC*-types. We observed losses of clades II and III and a drastic reduction of members from clades VI (two of seven members retained) and VII (one of five detected members retained) at week 8, while clades IV and IX diversified (Fig. 2). Six of the 19 universal *scaC*-types (types KW648P360, CC640P010, YH718P050 and JW648P071 (clade V), APT88P352 (clade VI), and HS648F36 (clade VII)) were not detected at week 8 and a further six universal types (groups US718F026, AFT80P016, AH640F088, RC710P054 and R640F023 (clade IV) and ADS80F316 (clade VII)) decreased dramatically between weeks 2 and 8. In contrast three of the universal *scaC*-types (groups ANH88F646 and ARF88P636 (clade IV) and TM710P666 (clade V)) dramatically increased in abundance over the same period with group ANH88F646 being detected only at week 8 (Fig. 2 and Table S6).

Few differences were seen among *scaC*-types between sample time post-feed, although universal *scaC*-type R640F023 (n = 8) was only detected at sampling periods 1 hour post-feed and JW648P071 (n = 9) was only detected at sampling periods 9 h post-feed even though both were detected in each animal at both weeks 2 and 5 (DM640P316 was also detected at week 8; Fig. 2).

**Figure 4:**
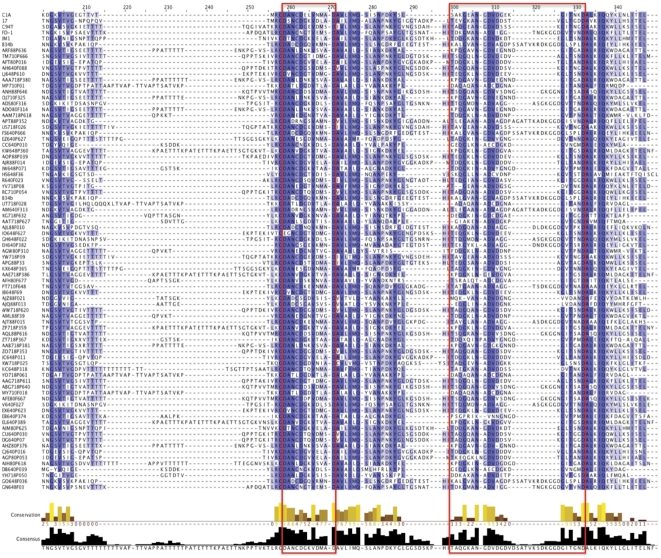
ClustalW alignments over the dockerin-containing region of representative sequences from the 75 observed metagenome and six reference *scaC*-types. The two repeats of the putative dockerin module are shown in red boxes. Blue coloring indicates sequence similarity with a darker shade indicating greater sequence conservation. Box plots of sequence conservation and conformation to a consensus sequence are also displayed along the bottom.

We then determined the potential extent of functional heterogeneity implicit within each *scaC*-type by comparing representative sequences. Among the *scaC*-types, we observed a high degree of amino acid sequence conservation at the N-terminus (Fig. S4), where the cohesin module is located [18]. Searches did confirm the presence of two characteristic repeats of a dockerin module in each *scaC*-type (Fig. 4).

The first dockerin repeat was strongly conserved amongst all sequences. However, the second repeat displayed much less sequence similarity among groups, although some amino acids commonly found in dockerin modules appeared strongly conserved throughout.

Collectively, these results show a great diversity of *scaC*-types present within the rumen ecosystem that may have functional connotations.

## Discussion

In this study we utilized the *scaC* gene as a probe to investigate the temporal and biogeographical intraspecific dynamics of *R. flavefaciens* within the rumen environment. In doing so, we were also able to evaluate the naturally occurring structural diversity of this important cellulosomal component.

The ∼1100 putative *scaC* sequences generated all contained modules with significant sequence similarity to dockerins and strong sequence conservation of the N-terminal-located cohesin. However, more diversity was observed in a second putative dockerin repeat, suggesting the genetic heterogeneity observed may have functional implications. Despite the genetic heterogeneity, the overall structure of the *scaC* sequences were consistent with the expected scaffoldin structure, thus validating the sequences generated as being true cellulosomal components.

The use of *scaC* as a gene marker [11] is consistent with previous efforts to study the population diversity of specialist microbial populations such as sulfate reducers [23], [24] and methanogenic archaea [25]–[28] by exploiting key genes critical to the species archetypical function. The use of *scaC* adds to this repertoire but with an important caveat being the confinement of *scaC* to a single ‘species’. With this in mind, our results suggest that there is a greater intraspecific genetic diversity of the *R. flavefaciens* species than previously recognized or seen in the current collection of reference strains.

Diversity estimates suggest that our survey provided information on approximately two-thirds of the total *scaC* diversity present. Yet, the *scaC*-population appeared skewed toward the nineteen *scaC*-types found in each rumen tested. Over the course of the survey, we observed differential enrichment of *scaC* populations between weeks, between animals and between fractions, but little difference was observed with respect to sampling time post-feeding. This suggests that the composition of *R. flavefaciens* populations do not fluctuate dramatically between feeding times.

There appeared some relationship between these dynamics and *scaC* phylogeny suggesting an adaptive evolutionary facet to these observations with functional implications. Clade VII, which includes *R. flavefaciens* FD-1, and Clade VIII were found almost exclusively in fiber-associated fractions, suggesting these *scaC*-types were more suited to insoluble substrate, or had a greater propensity to adhere.

Initially the temporal dynamics of *scaC* largely reflected those observed in the 16S rRNA gene population. However, by week 8 no significant separation of *scaC* populations was seen between animals suggesting a specific diet will ultimately enrich a constrained set of *scaC*-types. This was reflected in the relative enrichment and diversification of certain universal *scaC*-types and clades over the time course. In contrast, differences between animals are the largest separating factor of the 16S community. The enrichment of different microbial populations between liquid fractions and particulate matter correspondent to planktonic and fiber-adherent microbes has previously been reported through global 16S analysis [29]. Here we have shown this is also true within a species and is presumed to reflect the cellulosome-mediated fibrolytic function of *R. flavefaciens*.

Cellulosomes are attributed with having improved catalytic activities on crystalline substrates, as compared to individually acting enzymes, due to the improved synergy afforded by co-localization of complementary enzymatic activities [30]–[32]. Given this, it was interesting that certain *scaC*-types were enriched in planktonic fractions, and it will therefore be interesting to determine if these strains are more suited toward the deconstruction of soluble cellooligomers (C_2_–C_7_) and/or other soluble polysaccharides, while those enriched from fiber-adherent fractions are more suited to crystalline cellulose. It will also be interesting to determine if the relative abundances of *scaC*-groups bear any correlation with animal productivity and if this co-varies with diet.

Overall we have shown the use of *scaC* to be a convenient tool to survey the dynamics and intraspecific diversity of *R. flavefaciens* in an ecological context and suggest similar genetic-based approaches for the evaluation of other cellulolytic and specialist populations.

## Materials and Methods

### Ethics Statement

This study was approved by the Food/Fiber Institutional Animal Care and Use Committee (IACUC) with no stipulations and is filed with the IACUC approval number 04049.

### Rumen Sampling

Steers were maintained and fed as previously described [3]. Briefly, samples of whole rumen contents were obtained from three fistulated 5-yr old Angus Simmental Cross steers (samples 8, 64, and 71) that were maintained in open front barns at Illinois State University and housed singularly during the period of study. The steers were switched from a corn-silage diet (12.5% crude protein, 52% neutral detergent fiber (NDF-cellulose, hemicellulose and lignin), 39% acid detergent fiber (ADF-cellulose and lignin), 0.45% calcium, 0.32% phosphorus, 0.85% potassium, 0.18% sulfur) to a restricted diet of medium-quality grass-legume hay (13% crude protein, 60% neutral detergent fiber, 40% acid detergent fiber, 0.5% calcium, 0.25% phosphorus, and 0.5% trace mineralized salt) at maintenance intake based on 2001 National Research Council (NRC) nutrient recommendations for dairy cattle [33]. The animals were fed once a day for the entire length of the study (a total of eight weeks including two weeks prior to sampling). Approximately 3 L of whole rumen digesta (fiber-adherent and liquid associated microbes) were collected from the dorsal third rumen at week 2 (following dietary switch), week 5, and week 8, both one hour and nine hours after the morning feeding for a total of 36 samples. Samples were then partitioned into fiber-adherent fractions and liquid fractions prior to DNA extraction, using previously described methods [29], [34]. Samples were then stored at −80°C until DNA extraction.

### DNA Extraction and Purification

Genomic DNA was extracted using a protocol similar to the extraction of high molecular weight DNA for rumen and fecal contents [34]. Deviation from this protocol included following the Qiagen DNA Stool Kit manufacturer's protocol (Qiagen, Valencia, CA) after the addition of 960 µl of ASL buffer to the samples. DNA purity and concentration were analyzed by spectrophotometric quantification and gel electrophoresis.

### T-RFLP Analyses

All samples within the study were amplified using fluorescently labeled primers. Primers used for 16S analysis were 8F (5′NED-AGAGTTTGATCCTGGCTCAG-3′) and 926R (5′6FAM-CCGTCAATTCCTTTRAGTTT-3′) [35] while primers used for *scaC* gene analysis were ScaFwd (5′NED-AGACARGRTATAATHAAAGGGGC-3′) and ScaRev (5′6FAM-GGGTTTKTATTCCTTTGTAAG-3′) [11]. Amplified DNA was verified via gel-electrophoresis and spectrophotometric analyses prior to PCR purification (QiaQuick PCR purification Kit, Qiagen, Valencia, CA) and subsequent restriction enzymatic digestion. Enzymatic restriction digestions contained: 2 µl of 10× enzyme buffer (New England BioLabs, MA), 100 ng of PCR product, 20 µg of bovine serum albumin (New England BioLabs, MA), and 10 units of restriction enzymes (AluI, HhaI, MspI for 16S rDNA and AluI, HaeIII, MspI, and RsaI for scaC (New England Biolabs, Ipswich, MA)) for a total of 20 µl reactions. Reactions were incubated at 37°C (3 hr) and subsequently heat inactivated (20 min) at either 65°C for AluI, HhaI, MspI, and RsaI enzymes or 80°C for HaeIII. 1 µl aliquots were dried completely on 96-well plates using the Savant SpeedVac. Each sample was combined with 10 µl of a 1000∶15 mixture of HiDi Formamide (Applied Biosystems) and MapMarker 1000 (BioVentures, Inc.). Plates were denatured at 95°C (5 min) and cooled to 4°C (5 min) before loading onto the ABI 3730xl equipped with a 50-cm 96-capillary array and running POP-7 polymer software (Applied Biosystems). Samples were run using a modified version of the default GeneMapper50_POP7 run module, where the injection time was increased to 60 sec and run time increased to 3000 sec. Samples and ROX1000 standard (Applied Biosystems) were analyzed on GeneMapper v4.0 software (Applied Biosystems).

### Non-Metric Multidimensional Scaling Analyses

A comparison of 16S rDNA and *scaC* gene composition of all 36 rumen samples was conducted using non-metric multidimensional scaling (NMDS; [36]) analyses. Sample data were normalized prior to analyses [37]. In order to distinguish signal from noise for T-RFLP data, the total peak fluorescence was determined for each sample. After the sample with the lowest total peak fluorescence was determined, all other total peak heights of the remaining samples were normalized to generate an adjustment ratio. Each ratio, unique to each individual sample, was used to adjust all the peak heights within that sample to determine a new peak height. After completion, all peak heights below a cut-off of 25 fluorescence units (the theoretical limit of detection) were removed. Subsequently, new total peak heights were determined for each sample, and these new values were used to calculate corrected relative peak heights.

Fragment data were then normalized and subjected to an initial square-root transformation to reduce the emphasis on high abundance species and the Bray-Curtis similarity coefficient [38] was calculated for each possible pair of samples within either week 2, week 5, or week 8. This resulted in a 12-by-12 similarity matrix for each week and was used to conduct NMDS. NMDS was conducted using software Primer v6 for Windows [39], using 200 random starting configurations with the optimal two-dimensional solution having a final stress of 0. Statistical calculations and pair-wise comparisons were performed using one-way analysis of similarities (ANOSIM) for all factors (individual cow, hour sampled, fraction and week) generating R-coefficients and p-values for each comparison. ANOSIM was used to summarize differences between variable factors into a measure of dissimilarity. For example, a 100% dissimilarity measure (or an R-value of 1) indicates that for each group of a particular factor, there are no species in common. SIMPER was utilized to determine the relative contributions of each fragments to explaining group dissimilarities.

### Amplification of the scaC Gene for Cloning

Primers ScaFwd (5′-AGACARGRTATAATHAAAGGGGC-3′) and ScaRev (5′-GGGTTTKTATTCCTTTGTAAG-3′) were used for PCR amplification of the *scaC* gene [11]. Each PCR reaction contained: 1×Ex Taq buffer (Takara Bio Inc, WI), 0.25 mM dNTP mixture, 0.5 units Ex Taq™ DNA polymerase, 0.4 µM primer, 0.4 mg/ml bovine serum albumin, 1 µl of sample, and distilled water for a total of 25 µl reaction. Conditions for PCR amplification were 94°C (5 min), followed by 35 cycles of 94°C (30 sec), 51°C (30 sec), 72°C (1 min) and a final extension of 72°C (5 min). All PCR products were purified using QiaQuick PCR purification Kit (Qiagen, Valencia, CA, USA).

### Cloning and Sequencing of scaC Amplicons

Products from a *scaC*-directed PCR for all sampling time points were cloned into pGEMT-easy vector (Promega) and transformed by heat shock into competent *E. coli* JM109 (Promega). Transformants were selected by plating onto selective LB agar plates supplemented with 100 µg/ml ampicilin (LB/amp) that were incubated overnight. Colonies were picked randomly and re-streaked onto fresh LB/amp plates to ensure colonies were pure. Clean colonies were then grown overnight in 96-well culture plates containing LB/amp media supplemented with 8% glycerol. Plates were replicated into new glycerol stocks and then inoculated into 0.45 ml culture plates containing 2× LB and 100 µg/ml Carbenicillin. After 24 hr growth at 37°C, plasmid DNA was purified from the bacterial cultures using a heat-lysis protocol, where bacterial cultures were washed and then heated to 95°C. After centrifugation, the supernatant (containing plasmid DNA) was transferred to a new 96-well plate. Sequencing reactions were set up as follows: 5% glycerol, 1× Sequencing Buffer (Applied Biosystems), 0.1 µM sequencing primers (T7 promoter for 5′ reactions, SP6 for 3′ reactions), 2 µl of plasmid template, 0.25 µl of BigDye Terminator v3.1 (Applied Biosystems) and sterile distilled water in a 10 µl sample. Thermal cycling was performed at 96°C (5 min) followed by 35 cycles of 96°C (15 sec), 53°C (5 sec) and 60°C (4 min). When complete, reaction products were precipitated with 70 µl of 0.2 mM MgSO_4_ in 70% ethanol for 15 min and centrifuged at 3600 rpm (15 min). Plates were flipped onto paper towels and spun at 1000 rpm (1 min) to remove precipitation solution. The precipitated pellets were then dried for 10 min in the Savant SpeedVac to remove any residual ethanol. Samples were resuspended in 10 µl HiDi Formamide (Applied Biosystems), denatured at 95°C (5 min), and then loaded onto the ABI 3730xl equipped with a 50-cm 96-capillary array running POP-7 polymer (Applied Biosystems). Samples were run using a modified version of the default LongSeq50_POP7 run module, where injection time was increased to 25 sec and run time decreased to 5040 sec. Samples were analyzed on Sequencing Analysis v5.2 software (Applied Biosystems) for quality and then trimmed in Sequencher 4.5 to remove vector sequences. The resulting *scaC* sequences have been deposited with NCBI with accession numbers JN109234–JN110339.

### Sequence analysis

All *scaC* sequences were manually inspected and trimmed to their start and end codons using Artemis. Deduced ScaC amino acid sequences were aligned in ClustalX v 2.0 [40]. Dockerin modules were determined by the presence of their characteristic duplicated-22 amino acid repeat found within a 70 bp module, as previously described [13]. Rarefaction curves, richness estimators and diversity indices were generated in DOTUR [22] using Fastgroup II [20]. Maximum likelihood trees were created using the default parameters of RAxML version 7.0.4 [21] for rapid bootstrap and maximum likelihood searches. The resulting tree was visualized in Archaeopteryx viewer [41].

The nucleotide sequences of *scaC* were clustered using FastGroupII (http://biome.sdsu.edu/fastgroup; [20]) at various sequence similarities. The *scaC*-types were labeled by the first binned clone id, except for the group clustering with *R. flavefaciens* B34b, which was named after the reference strain. Alignments of the representative and remaining sequences within each group were made using the ‘slow and accurate’ parameters in ClustalX [40]. Alignments were viewed and curated using Jalview [42].

## Supporting Information

Figure S1 NMDS plots of 16S T-RFLP analyses using restriction enzymes (A) *Alu*I or (B) *Hha*I. Weeks are indicated at top left of each panel. Numerical representations indicate bovine number (8, 64, or 71) and symbols indicate either fiber-adherent fraction (green **×**) or liquid fraction (blue ▾).
(TIF)

Figure S2 NMDS plots of ScaC T-RFLP analyses using restriction enzymes (A) *Alu*I, (B) *Hae*III, or (C) *Msp*I. Weeks are indicated at top right of each panel. Numerical representations above symbols indicate bovine number (8, 64, or 71) and symbols indicate either fiber-adherent (green **×**) or liquid fraction (blue ▾).
(TIF)

Figure S3 **Overall ScaC alignments sorted by** ***scaC*** **-type.** The amino acid sequence of up to fifteen randomly selected sequences from each of the most abundant *scaC*-types is shown. Blue panels indicate overall sequence conservation. Blue coloring indicates degree of sequence conservation with a darker shade tending toward 100% ID. The location and identities of *scaC*-types shown are indicated.
(TIF)

Figure S4 Full ClustalW alignments of the 75 representative ScaC sequences.
(TIF)

Table S1 Taxonomic results of T-RFLP fragment profile to *in silico* digests of rumen libraries using phylogenetic assessment tool (PAT) [18].
(DOC)

Table S2 Similarity percentage analysis (SIMPER) by fraction in *scaC* fragment profiles. The top 10 contributing species fragments and their percent contributions to the total fragment abundance are listed at each week of the study (0, 3, and 6) for enzymes *Alu*I (A), *Hae*III (B), *Msp*I (C), and *Rsa*I (D). Fragments highlighted are present within each week using that particular restriction enzyme. Additionally, the average dissimiliarity percentages between the fiber-adherent and liquid fraction is listed for each sampling week.
(DOC)

Table S3 Similarity percentage analysis (SIMPER) by fraction in 16S rRNA fragment profiles. The top 10 contributing species fragments and their percent contributions to the total fragment abundance are listed at each week of the study (0, 3, and 6) for enzymes *Alu*I(A), *Hha*I(B), and *Msp*I(C). Fragments highlighted are present within each week using that particular restriction enzyme. Additionally, the average dissimiliarity percentages between the fiber-adherent and liquid fraction is listed for each sampling week.
(DOC)

Table S4 ScaC-types and the number of sequences clustering at 70% amino acid identity within each group by animal and overall. Those groups that cluster with *R. flavefaciens* reference strains are indicated in bold. Group names were assigned based on the first sequence binned to that group.
(DOC)

Table S5 The percent abundances of the nineteen universal *scaC*-types arranged by rumen sampled. The proportion of sequences obtained that clustered with each *scaC*-type is shown. Their relative abundance overall is also shown.
(DOC)

Table S6 The relative distribution of universal *scaC*-types between sample points post feed, fraction or sample week. All values have been normalized to the total number of reads acquired for each sample variable.
(DOC)
