# Supplementary figures and images for: Cellulosomics, a Gene-Centric Approach to Investigating the Intraspecific Diversity and Adaptation of Ruminococcus flavefaciens within the Rumen
Source: PLoS One. 2011 Oct 17;6(10):e25329. doi: 10.1371/journal.pone.0025329 (PMC3197198; doi:10.1371/journal.pone.0025329)

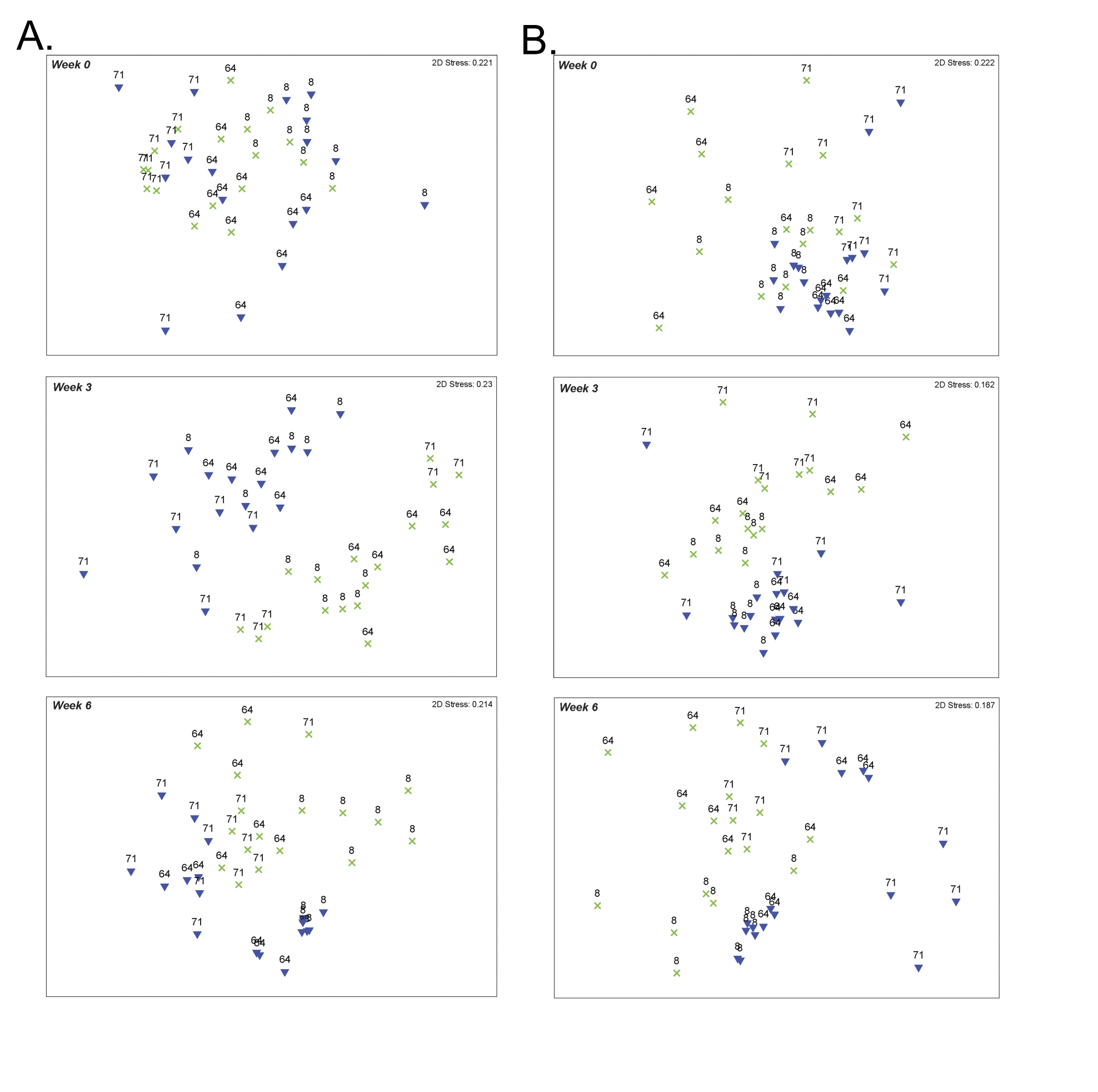

Supplement: Figure S1 — NMDS plots of 16S T-RFLP analyses using restriction enzymes (A) AluI or (B) HhaI. Weeks are indicated at top left of each panel. Numerical representations indicate bovine number (8, 64, or 71) and symbols indicate either fiber-adherent fraction (green ×) or liquid fraction (blue ▾). (TIF) [file pone.0025329.s001.tif]

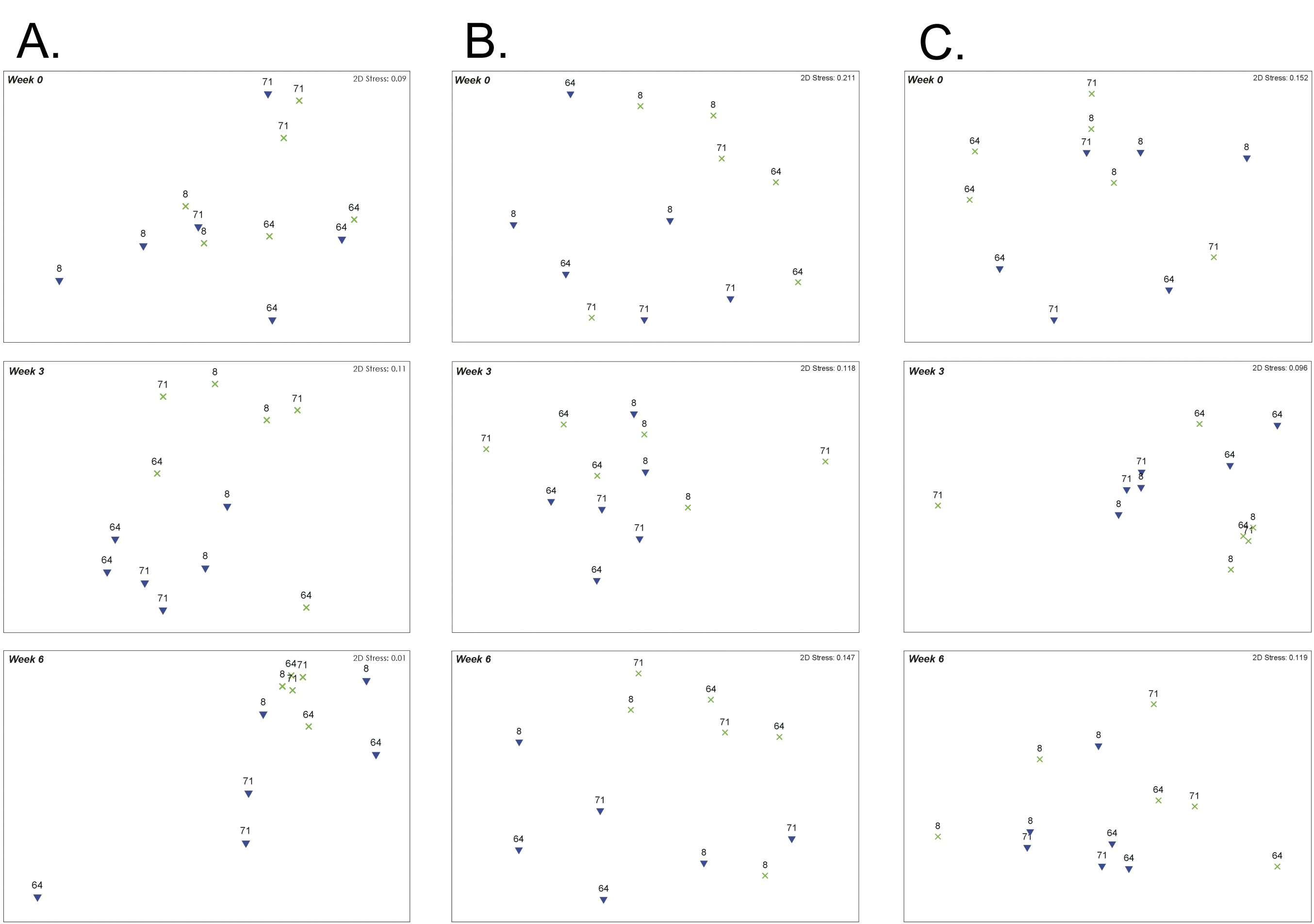

Supplement: Figure S2 — NMDS plots of ScaC T-RFLP analyses using restriction enzymes (A) AluI, (B) HaeIII, or (C) MspI. Weeks are indicated at top right of each panel. Numerical representations above symbols indicate bovine number (8, 64, or 71) and symbols indicate either fiber-adherent (green ×) or liquid fraction (blue ▾). (TIF) [file pone.0025329.s002.tif]

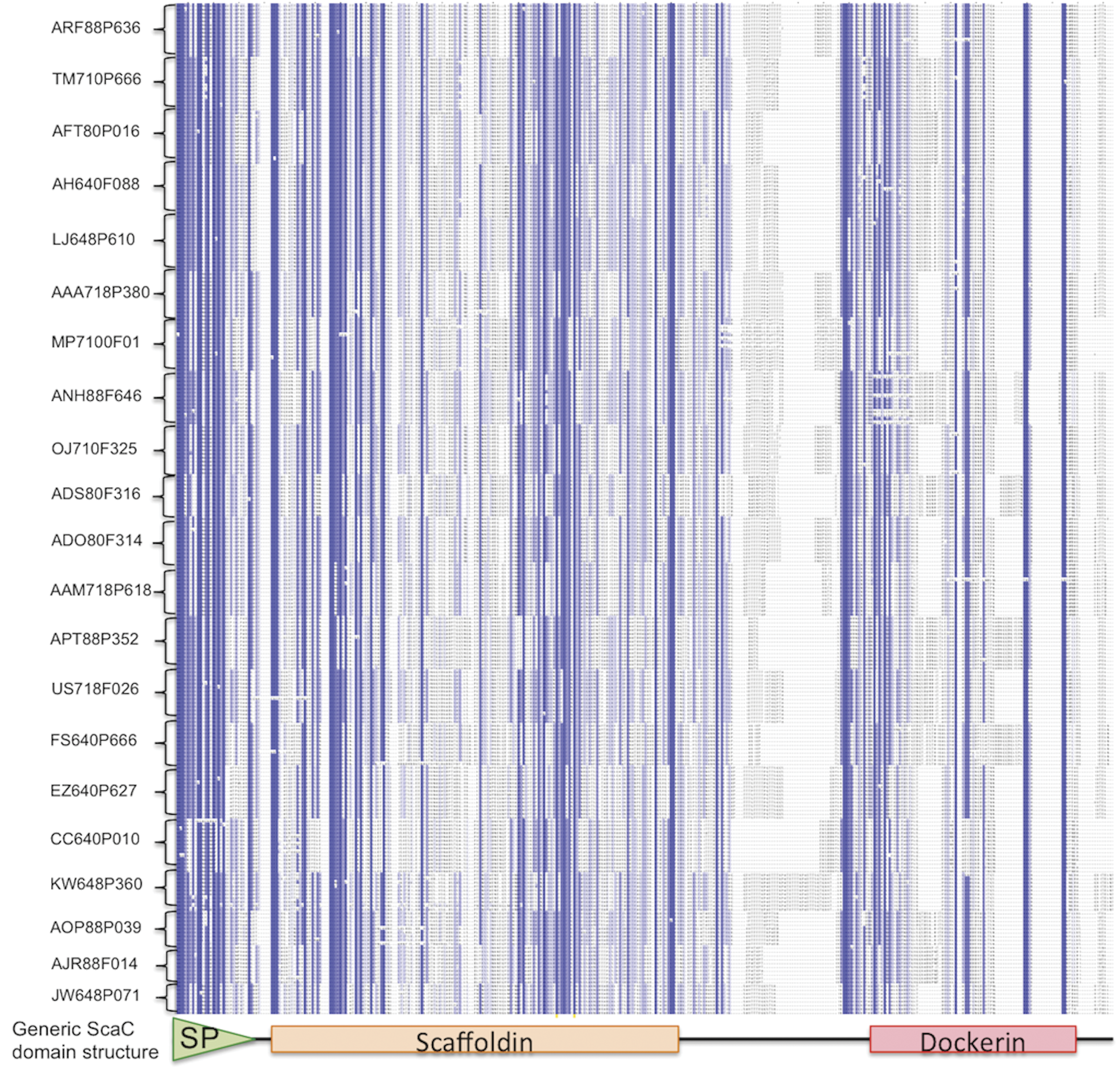

Supplement: Figure S3 — Overall ScaC alignments sorted by scaC -type. The amino acid sequence of up to fifteen randomly selected sequences from each of the most abundant scaC-types is shown. Blue panels indicate overall sequence conservation. Blue coloring indicates degree of sequence conservation with a darker shade tending toward 100% ID. The location and identities of scaC-types shown are indicated. (TIF) [file pone.0025329.s003.tif]

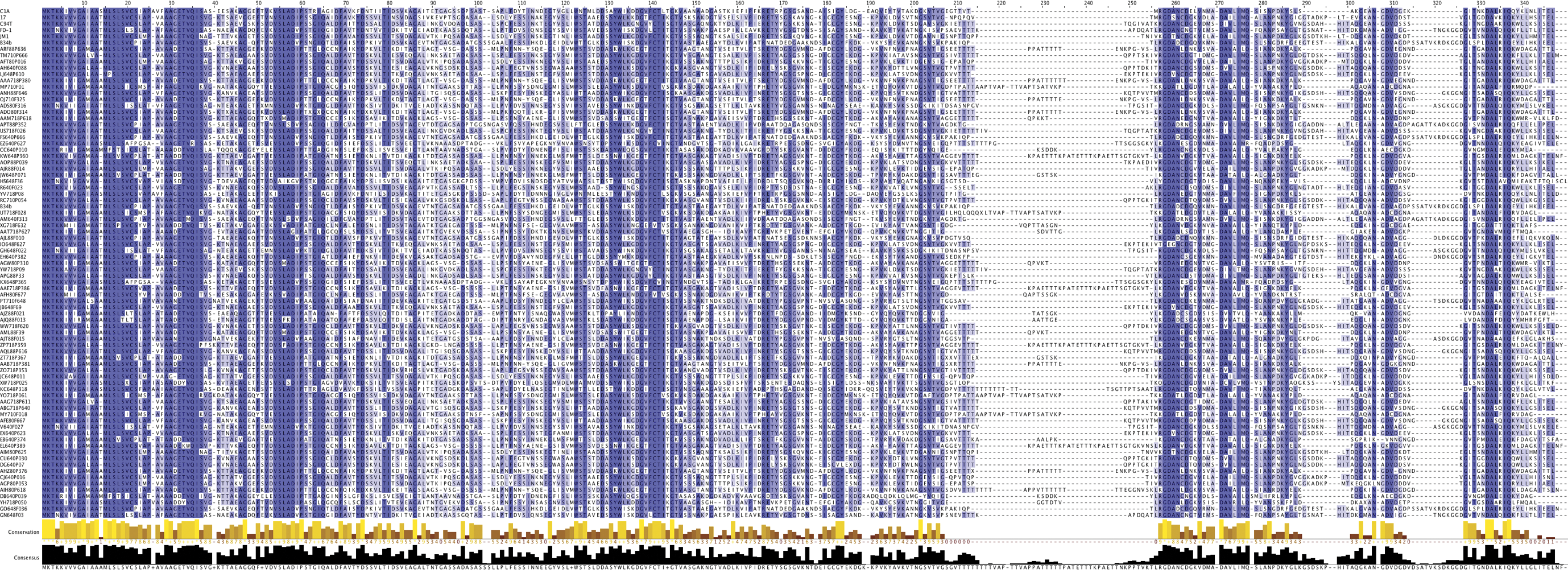

Supplement: Figure S4 — Full ClustalW alignments of the 75 representative ScaC sequences. (TIF) [file pone.0025329.s004.tif]
