# Supplementary material for: Cellulosomics, a Gene-Centric Approach to Investigating the Intraspecific Diversity and Adaptation of Ruminococcus flavefaciens within the Rumen
Source: PLoS One. 2011 Oct 17;6(10):e25329. doi: 10.1371/journal.pone.0025329 (PMC3197198; doi:10.1371/journal.pone.0025329)
Supplement: Table S1 — Taxonomic results of T-RFLP fragment profile to in silico digests of rumen libraries using phylogenetic assessment tool (PAT) [18]. (DOC) [file pone.0025329.s005.doc]

|  | **Enzyme** | | |
| --- | --- | --- | --- |
|  | ***Alu*I** | ***Hha*I** | ***Msp*I** |
| Total number of peaks | 2628 | 2511 | 6079 |
| Average fragments per sample | 24 | 23 | 57 |
| Max. fragments per sample | 83 | 101 | 121 |
| Min. fragments per sample | 6 | 4 | 15 |
| Fragments classified by Phylum: | | | |
| *Bacteroidetes* | 17 (0.7 %) | 90 (3.6 %) | 73 (1.2 %) |
| *Firmicutes* | 672 (25.6 %) | 988 (39.4 %) | 2731 (44.9 %) |
| *Proteobacteria* | 0 | 9 (0.4%) | 8 (0.1 %) |
| *TM7* | 1 (0.04 %) | 0 | 0 |
| Total percent classified: | 26.26 % | 43.29 % | 46.26 % |
| Undetermined fragments | 1938 (73.7 %) | 1424 (56.7 %) | 3267 (53.7 %) |
| Unmatched fragments | 341 (13.0 %) | 467 (18.6 %) | 635 (10.5 %) |
