# Supplementary material for: Cellulosomics, a Gene-Centric Approach to Investigating the Intraspecific Diversity and Adaptation of Ruminococcus flavefaciens within the Rumen
Source: PLoS One. 2011 Oct 17;6(10):e25329. doi: 10.1371/journal.pone.0025329 (PMC3197198; doi:10.1371/journal.pone.0025329)
Supplement: Table S3 — Similarity percentage analysis (SIMPER) by fraction in 16S rRNA fragment profiles. The top 10 contributing species fragments and their percent contributions to the total fragment abundance are listed at each week of the study (0, 3, and 6) for enzymes AluI(A), HhaI(B), and MspI(C). Fragments highlighted are present within each week using that particular restriction enzyme. Additionally, the average dissimiliarity percentages between the fiber-adherent and liquid fraction is listed for each sampling week. (DOC) [file pone.0025329.s007.doc]

| ***Alu*I** | | | | | | | | | | | |
| --- | --- | --- | --- | --- | --- | --- | --- | --- | --- | --- | --- |
| Week 0 | | | | Week 3 | | |  | | Week 6 | | |
| Frag Length |  | Percent |  | Frag Length |  | Percent |  | | Frag Length |  | Percent |
| 284 |  | 4.52 |  | 244 |  | 3.54 |  | | 256 |  | 2.79 |
| 252 |  | 3.17 |  | 71 |  | 2.67 |  | | 284 |  | 2.72 |
| 285 |  | 2.94 |  | 601 |  | 2.37 |  | | 245 |  | 1.99 |
| 228 |  | 2.52 |  | 757 |  | 2.16 |  | | 76 |  | 1.82 |
| 222 |  | 2.31 |  | 284 |  | 2.09 |  | | 757 |  | 1.82 |
| 241 |  | 2.25 |  | 800 |  | 1.89 |  | | 222 |  | 1.75 |
| 240 |  | 2.22 |  | 245 |  | 1.85 |  | | 228 |  | 1.73 |
| 750 |  | 2.22 |  | 750 |  | 1.71 |  | | 253 |  | 1.64 |
| 72 |  | 2.17 |  | 700 |  | 1.63 |  | | 72 |  | 1.63 |
| 245 |  | 2.14 |  | 222 |  | 1.54 |  | | 455 |  | 1.59 |
| Average dissimilarity: | | 77.72% |  |  |  | 90.39% |  | |  |  | 73.85% |
| ***Hha*I** | | | | | | | | | | | |
| 131 |  | 2.81 |  | 99 |  | 7.6 |  | 99 | |  | 3.2 |
| 371 |  | 2.74 |  | 224 |  | 3.28 |  | 575 | |  | 3.15 |
| 82 |  | 2.67 |  | 62 |  | 3.01 |  | 91 | |  | 2.59 |
| 575 |  | 2.66 |  | 575 |  | 2.7 |  | 191 | |  | 2.36 |
| 91 |  | 2.66 |  | 140 |  | 2.62 |  | 131 | |  | 2.26 |
| 850 |  | 2.27 |  | 91 |  | 2.52 |  | 576 | |  | 1.83 |
| 650 |  | 2.26 |  | 576 |  | 2.42 |  | 371 | |  | 1.8 |
| 272 |  | 2.19 |  | 168 |  | 2.32 |  | 62 | |  | 1.74 |
| 750 |  | 1.99 |  | 750 |  | 2.29 |  | 236 | |  | 1.45 |
| 700 |  | 1.96 |  | 131 |  | 2.22 |  | 750 | |  | 1.39 |
| Average dissimilarity: | | 69.43% |  |  |  | 88.45% |  |  | |  | 84.01% |
| ***Msp*I** | | | | | | | | | | | |
| 137 |  | 2.12 |  | 93 |  | 4.31 |  | 93 | |  | 1.58 |
| 135 |  | 2.05 |  | 137 |  | 2.55 |  | 236 | |  | 1.57 |
| 93 |  | 1.96 |  | 201 |  | 2.16 |  | 237 | |  | 1.56 |
| 142 |  | 1.81 |  | 89 |  | 1.84 |  | 147 | |  | 1.35 |
| 223 |  | 1.42 |  | 496 |  | 1.61 |  | 138 | |  | 1.3 |
| 147 |  | 1.41 |  | 138 |  | 1.54 |  | 131 | |  | 1.27 |
| 295 |  | 1.4 |  | 131 |  | 1.51 |  | 142 | |  | 1.26 |
| 131 |  | 1.38 |  | 147 |  | 1.28 |  | 68 | |  | 1.21 |
| 148 |  | 1.37 |  | 486 |  | 1.2 |  | 216 | |  | 1.2 |
| 216 |  | 1.26 |  | 87 |  | 1.17 |  | 137 | |  | 1.17 |
| Average dissimilarity: | | 52.51% |  |  |  | 80.82% |  |  | |  | 57.79% |
