# Supplementary material for: Cellulosomics, a Gene-Centric Approach to Investigating the Intraspecific Diversity and Adaptation of Ruminococcus flavefaciens within the Rumen
Source: PLoS One. 2011 Oct 17;6(10):e25329. doi: 10.1371/journal.pone.0025329 (PMC3197198; doi:10.1371/journal.pone.0025329)
Supplement: Table S4 — ScaC-types and the number of sequences clustering at 70% amino acid identity within each group by animal and overall. Those groups that cluster with R. flavefaciens reference strains are indicated in bold. Group names were assigned based on the first sequence binned to that group. (DOC) [file pone.0025329.s008.doc]

| ***scaC*-type** | **Rumen 8** | **Rumen 64** | **Rumen 71** | **Total** |
| --- | --- | --- | --- | --- |
| ARF88P636 | 126 | 14 | 23 | 163 |
| TM710P666 | 31 | 35 | 73 | 139 |
| AFT80P016 | 68 | 13 | 27 | 108 |
| AH640F088 | 32 | 35 | 23 | 90 |
| LJ648P610 | 0 | 75 | 11 | 86 |
| AAA718P380 | 7 | 22 | 31 | 60 |
| MP710F01 | 0 | 1 | 48 | 49 |
| ANH88F646 | 34 | 2 | 6 | 42 |
| OJ710F325 | 0 | 2 | 40 | 42 |
| ADS80F316 | 16 | 10 | 9 | 35 |
| ADO80F314 | 22 | 0 | 2 | 24 |
| AAM718P618 | 16 | 2 | 4 | 22 |
| APT88P352 | 6 | 3 | 10 | 19 |
| US718F026 | 2 | 5 | 12 | 19 |
| FS640P666 | 0 | 19 | 0 | 19 |
| EZ640P627 | 0 | 6 | 10 | 16 |
| CC640P010 | 1 | 10 | 3 | 14 |
| KW648P360 | 2 | 10 | 1 | 13 |
| AOP88P039 | 4 | 1 | 5 | 10 |
| AJR88F014 | 5 | 2 | 3 | 10 |
| JW648P071 | 3 | 5 | 1 | 9 |
| HS648F36 | 1 | 7 | 1 | 9 |
| R640F023 | 1 | 6 | 1 | 8 |
| YV718P08 | 5 | 0 | 3 | 8 |
| RC710P054 | 1 | 2 | 4 | 7 |
| YH718P050 | 2 | 1 | 2 | 5 |
| GN648F03 | 0 | 4 | 1 | 5 |
| B34b | 0 | 5 | 0 | 5 |
| UT718F028 | 0 | 1 | 4 | 5 |
| AM640F313 | 0 | 3 | 2 | 5 |
| XG718F632 | 0 | 0 | 4 | 4 |
| AAT718P627 | 0 | 0 | 2 | 2 |
| AJL88F010 | 1 | 1 | 0 | 2 |
| IO648F627 | 0 | 2 | 0 | 2 |
| GH648F022 | 0 | 1 | 1 | 2 |
| EH640P382 | 0 | 2 | 0 | 2 |
| AGW80P310 | 1 | 0 | 1 | 2 |
| YW718P09 | 0 | 1 | 1 | 2 |
| APG88P33 | 2 | 0 | 0 | 2 |
| KX648P365 | 0 | 1 | 1 | 2 |
| AAE718P386 | 1 | 0 | 1 | 2 |
| AFH80F677 | 1 | 0 | 1 | 2 |
| PT710F648 | 0 | 1 | 1 | 2 |
| JB648F69 | 0 | 1 | 0 | 1 |
| AJZ88F021 | 1 | 0 | 0 | 1 |
| AJQ88F013 | 1 | 0 | 0 | 1 |
| WW718F620 | 0 | 0 | 1 | 1 |
| AML88F39 | 1 | 0 | 0 | 1 |
| AJT88F015 | 1 | 0 | 0 | 1 |
| ZP718P359 | 0 | 0 | 1 | 1 |
| AQL88P616 | 1 | 0 | 0 | 1 |
| ZT718P367 | 0 | 0 | 1 | 1 |
| AAB718P381 | 0 | 0 | 1 | 1 |
| ZO718P353 | 0 | 0 | 1 | 1 |
| JC648P011 | 0 | 1 | 0 | 1 |
| XW718P025 | 0 | 0 | 1 | 1 |
| KC648P318 | 0 | 1 | 0 | 1 |
| YO718P061 | 0 | 0 | 1 | 1 |
| AAG718P611 | 0 | 0 | 1 | 1 |
| ABG718P640 | 0 | 0 | 1 | 1 |
| MY710F018 | 0 | 0 | 1 | 1 |
| AFE80F667 | 1 | 0 | 0 | 1 |
| V640F027 | 0 | 1 | 0 | 1 |
| EX640P623 | 0 | 1 | 0 | 1 |
| EB640P374 | 0 | 1 | 0 | 1 |
| EL640P389 | 0 | 1 | 0 | 1 |
| AIM80P625 | 1 | 0 | 0 | 1 |
| CU640P030 | 0 | 1 | 0 | 1 |
| DG640P07 | 0 | 1 | 0 | 1 |
| AHZ80P376 | 1 | 0 | 0 | 1 |
| CJ640P016 | 0 | 1 | 0 | 1 |
| AGP80P053 | 1 | 0 | 0 | 1 |
| AIH80P618 | 1 | 0 | 0 | 1 |
| DB640P039 | 0 | 1 | 0 | 1 |
| ***TOTAL*** | ***401*** | ***321*** | ***383*** | ***1106*** |
