# Supplementary material for: Cellulosomics, a Gene-Centric Approach to Investigating the Intraspecific Diversity and Adaptation of Ruminococcus flavefaciens within the Rumen
Source: PLoS One. 2011 Oct 17;6(10):e25329. doi: 10.1371/journal.pone.0025329 (PMC3197198; doi:10.1371/journal.pone.0025329)
Supplement: Table S5 — The percent abundances of the nineteen universal scaC-types arranged by rumen sampled. The proportion of sequences obtained that clustered with each scaC-type is shown. Their relative abundance overall is also shown. (DOC) [file pone.0025329.s009.doc]

| **Sample**  **/ *scaC*-type** | **Rumen 8** | **Rumen 64** | **Rumen 71** | **Overall** |
| --- | --- | --- | --- | --- |
| ARF88P636 | 31.42 | 4.36 | 6.01 | 14.74 |
| TM710P666 | 7.73 | 10.90 | 19.06 | 12.57 |
| AFT80P016 | 16.96 | 4.05 | 7.05 | 9.76 |
| AH640F088 | 7.98 | 10.90 | 6.01 | 8.14 |
| AAA718P380 | 1.75 | 6.85 | 8.09 | 5.42 |
| ANH88F646 | 8.48 | 0.62 | 1.57 | 3.80 |
| ADS80F316 | 3.99 | 3.12 | 2.35 | 3.16 |
| AAM718P618 | 3.99 | 0.62 | 1.04 | 1.99 |
| APT88P352 | 1.50 | 0.93 | 2.61 | 1.72 |
| US718F026 | 0.50 | 1.56 | 3.13 | 1.72 |
| CC640P010 | 0.25 | 3.12 | 0.78 | 1.27 |
| KW648P360 | 0.50 | 3.12 | 0.26 | 1.18 |
| AOP88P039 | 1.00 | 0.31 | 1.31 | 0.90 |
| AJR88F014 | 1.25 | 0.62 | 0.78 | 0.90 |
| JW648P071 | 0.75 | 1.56 | 0.26 | 0.81 |
| HS648F36 | 0.25 | 2.18 | 0.26 | 0.81 |
| R640F023 | 0.25 | 1.87 | 0.26 | 0.72 |
| RC710P054 | 0.25 | 0.62 | 1.04 | 0.63 |
| YH718P050 | 0.50 | 0.31 | 0.52 | 0.45 |
| ***TOTAL*** | **89.28** | **57.63** | **62.40** | **70.71** |
