# Supplementary material for: Cellulosomics, a Gene-Centric Approach to Investigating the Intraspecific Diversity and Adaptation of Ruminococcus flavefaciens within the Rumen
Source: PLoS One. 2011 Oct 17;6(10):e25329. doi: 10.1371/journal.pone.0025329 (PMC3197198; doi:10.1371/journal.pone.0025329)
Supplement: Table S6 — The relative distribution of universal scaC-types between sample points post feed, fraction or sample week. All values have been normalized to the total number of reads acquired for each sample variable. (DOC) [file pone.0025329.s010.doc]

|  | **Time post feed** | | **Fraction** | | **Week** | | |
| --- | --- | --- | --- | --- | --- | --- | --- |
| ***scaC*-type** | **1 hr** | **8 hr** | **Planktonic** | **Fiber** | **0** | **3** | **6** |
| ARF88P636 | 46.24 | 53.76 | 54.72 | 45.28 | 0.53 | 57.24 | 42.24 |
| TM710P666 | 46.99 | 53.01 | 57.79 | 42.21 | 9.55 | 41.95 | 48.50 |
| AFT80P016 | 68.71 | 31.29 | 47.65 | 52.35 | 82.08 | 10.28 | 7.63 |
| AH640F088 | 54.67 | 45.33 | 63.99 | 36.01 | 84.83 | 8.27 | 6.90 |
| AAA718P380 | 41.89 | 58.11 | 67.90 | 32.10 | 19.14 | 46.61 | 34.25 |
| ANH88F646 | 43.08 | 56.92 | 28.17 | 71.83 | 0 | 0 | 100 |
| ADS80F316 | 63.07 | 36.93 | 0 | 100 | 54.51 | 42.74 | 2.75 |
| AAM718P618 | 41.13 | 58.87 | 54.05 | 45.95 | 19.30 | 68.45 | 12.25 |
| APT88P352 | 47.59 | 52.41 | 78.61 | 21.39 | 17.39 | 82.61 | 0 |
| US718F026 | 52.86 | 47.14 | 46.87 | 53.13 | 58.68 | 30.97 | 10.34 |
| CC640P010 | 35.92 | 64.08 | 49.50 | 50.50 | 91.12 | 8.88 | 0 |
| KW648P360 | 30.96 | 69.04 | 100 | 0 | 0 | 100 | 0 |
| AOP88P039 | 10.08 | 89.92 | 59.52 | 40.48 | 36.45 | 34.63 | 28.92 |
| AJR88F014 | 40.22 | 59.78 | 39.52 | 60.48 | 57.47 | 12.13 | 30.39 |
| JW648P071 | 0 | 100 | 88.69 | 11.31 | 61.22 | 38.78 | 0 |
| HS648F36 | 33.53 | 66.47 | 10.92 | 89.08 | 49.67 | 50.33 | 0 |
| R640F023 | 100 | 0 | 74.63 | 25.37 | 58.20 | 29.49 | 12.31 |
| RC710P054 | 57.36 | 42.64 | 71.02 | 28.98 | 85.01 | 0 | 14.99 |
| YH718P050 | 20.15 | 79.85 | 59.52 | 40.48 | 75.95 | 24.05 | 0 |
| **Other groups** | **51.0** | **49.0** | **41.80** | **58.20** | **33.05** | **23.79** | **43.16** |
